# Loss of CEP70 function affects acrosome biogenesis and flagella formation during spermiogenesis

**DOI:** 10.1038/s41419-021-03755-z

**Published:** 2021-05-12

**Authors:** Qiang Liu, Qianying Guo, Wei Guo, Shi Song, Nan Wang, Xi Chen, Andi Sun, Liying Yan, Jie Qiao

**Affiliations:** 1 Center for Reproductive Medicine, Department of Obstetrics and Gynecology, Peking University Third Hospital, Beijing, China; 2 National Clinical Research Center for Obstetrics and Gynecology (Peking University Third Hospital), Beijing, China; 3 Key Laboratory of Assisted Reproduction (Peking University), Ministry of Education, Beijing, China; 4 Beijing Key Laboratory of Reproductive Endocrinology and Assisted Reproductive Technology, Beijing, China; 5 Research Units of Comprehensive Diagnosis and Treatment of Oocyte Maturation Arrest, Chinese Academy of Medical Sciences, Beijing, China

**Keywords:** Apoptosis, Infertility

## Abstract

The spermatogenesis process is complex and delicate, and any error in a step may cause spermatogenesis arrest and even male infertility. According to our previous transcriptomic data, *CEP70* is highly expressed throughout various stages of human spermatogenesis, especially during the meiosis and deformation stages. CEP70 is present in sperm tails and that it exists in centrosomes as revealed by human centrosome proteomics. However, the specific mechanism of this protein in spermatogenesis is still unknown. In this study, we found a heterozygous site of the same mutation on *CEP70* through mutation screening of patients with clinical azoospermia. To further verify, we deleted CEP70 in mice and found that it caused abnormal spermatogenesis, leading to male sterility. We found that the knockout of CEP70 did not affect the prophase of meiosis I, but led to male germ-cell apoptosis and abnormal spermiogenesis. By transmission electron microscopy (TEM) and scanning electron microscopy (SEM) analysis, we found that the deletion of CEP70 resulted in the abnormal formation of flagella and acrosomes during spermiogenesis. Tandem mass tag (TMT)-labeled quantitative proteomic analysis revealed that the absence of CEP70 led to a significant decrease in the proteins associated with the formation of the flagella, head, and acrosome of sperm, and the microtubule cytoskeleton. Taken together, our results show that CEP70 is essential for acrosome biogenesis and flagella formation during spermiogenesis.

## Introduction

Infertility is a serious health problem worldwide. According to the latest data from the World Health Organization (WHO), around 10–15% of couples (50–80 million people) of reproductive age are suffering from infertility. Male factors account for about 50% of these cases of infertility^1–3^. In addition, recent data showed that the male etiology of ~70% of infertile couples remains unexplained^4^. In male infertility patients, there is a type of disease called oligoasthenoteratozoospermia (OAT) that is mainly characterized by a decreased sperm concentration and motility, and a higher rate of abnormal shapes^5^. As a key cause of male infertility, azoospermia or OAT is mainly caused by severe damage to spermatogenesis^6^. Spermatogenesis is a complicated process that involves the proliferation of spermatogonial stem cells (SSCs), meiosis of spermatocytes, haploid differentiation, and spermiogenesis^7,8^. In the process of spermiogenesis, round spermatids undergo a series of dynamic morphological changes, including the formation of acrosomes, mitochondrial sheath and flagella, and cytoplasmic clearance^9–11^. The transcriptomic analyses have shown that there are more than 4000 genes involved in the process of human spermatogenesis^12^. Therefore, many unknown genetic factors that affect spermatogenesis require further investigation.

According to our previous transcriptomic data, *CEP70*, which belongs to the centrosome protein family (CEP family), is highly expressed throughout various stages of human spermatogenesis, especially during the meiosis and deformation stages^13^. Additionally, analysis of the reported proteomic data of human sperm tails found that CEP70 is present in sperm tails^14^. As a centrosome protein, CEP70, which was first discovered in human centrosome proteomics, is located in the centrosome throughout the cell cycle and interacts with γ-tubulin by two coiled-coil domains^15,16^. Within the CEP family, studies have shown that CEP135 and CEP131 are involved in spermatogenesis^17,18^. Moreover, CEP70 participates in the extension and dynamic regulation of microtubules and interacts with histone deacetylase 6 (HDAC6) to regulate the stability of microtubules^19,20^. In zebrafish, knocking down *cep70* and *cep131* can cause abnormal cilia production^21^. Based on these data, we hypothesize that CEP70 may play important roles in regulating mammalian spermatogenesis and its mutation is related to male infertility.

In this study, we conducted mutation screening of patients with clinical azoospermia and found a heterozygous site of the same mutation on *CEP70*. To further verify, we deleted CEP70 in mice and found that it caused abnormal spermiogenesis, leading to male sterility. Finally, we used quantitative proteomic analysis to revealed that the absence of CEP70 led to a significant decrease in the proteins associated with the formation of the flagella, head, and acrosome of sperm, and the microtubule cytoskeleton.

## Results

### CEP70 may play an important function in human spermatogenesis

**Fig. 1:**
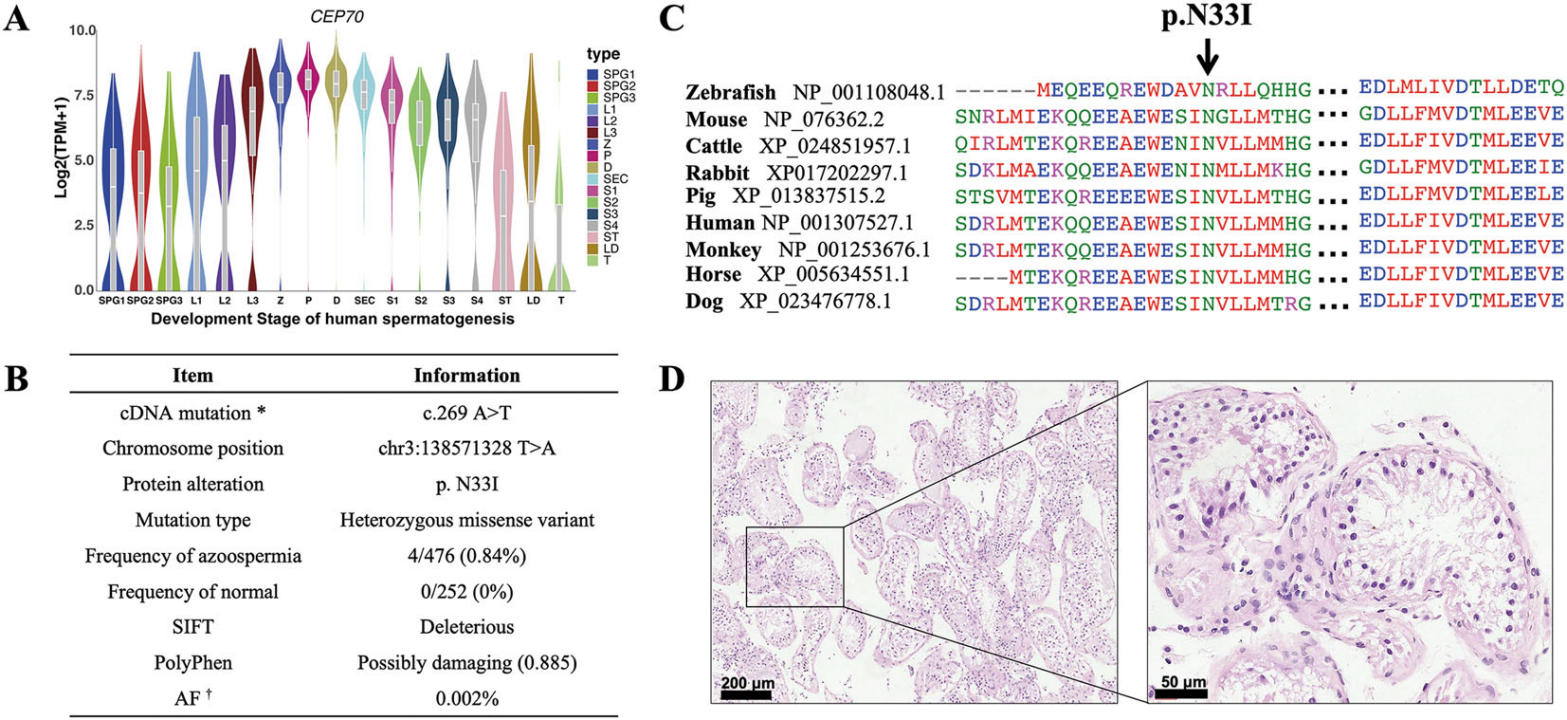
The expression pattern of *CEP70* during human spermatogenesis and clinical mutation screening. **A** The mRNA expression pattern of *CEP70* during human spermatogenesis. SPG1: spermatogonial stem cells; SPG2: differentiating spermatogonia; SPG3: differentiated spermatogonia; L1-L3: three consecutive stages of leptotene spermatocytes; Z: zygotene; P: pachytene; D: diplotene; SEC: a mixture of diakinesis, metaphase, anaphase, telophase and secondary spermatocytes; S1–S4: four stages of spermatids; ST: Sertoli cell; LD: peritubular myoid cells (PMCs) and Leydig cells; T: testicular macrophages. **B** Analysis of *CEP70* mutation in azoospermia patients. *: the accession number for CEP70 is GenBank: NM_024491.4; †: frequency of existing variant in 1000 Genomes combined population. **C** Homology analysis of amino acid changes caused by mutation sites among different species. **D** H&E staining of clinicopathological sections of azoospermia patients with the same heterozygous mutation.

According to the single-cell transcriptome data of germ cells^13^, *CEP70* is highly expressed in various stages of human spermatogenesis, especially during the meiosis and deformation stages (Fig. 1A). To determine whether the *CEP70* mutation is associated with clinical azoospermia patients, we designed 14 pairs of primers (listed in Supplementary Table S2) for the coding sequence of *CEP70* to investigate the variation of it in 476 infertile patients with azoospermia and 252 men with normal fertility. After sequencing, we found that four subjects with azoospermia had the same heterozygous mutation site (c.269A>T, p. N33I), while there was no such mutation in the control group (Fig. 1B). This mutation was conserved among various species (Fig. 1C) and predicted to be deleterious and possibly damaging by SIFT and PolyPhen2 (https://asia.ensembl.org/Multi/Tools/VEP). Through the clinical pathology report, it was found that two of the four cases had Sertoli cell-only syndrome (SCO), which caused azoospermia (Fig. 1D). However, there were no homozygous mutations or compound heterozygous mutations were found in the azoospermia patients, indicating that the recessive CEP70 may just relate to azoospermia and the deleterious heterozygous mutation of *CEP70* may increase the risk of azoospermia.

### Knockout of *Cep70* causes spermatogenesis disorders and male infertility

**Fig. 2:**
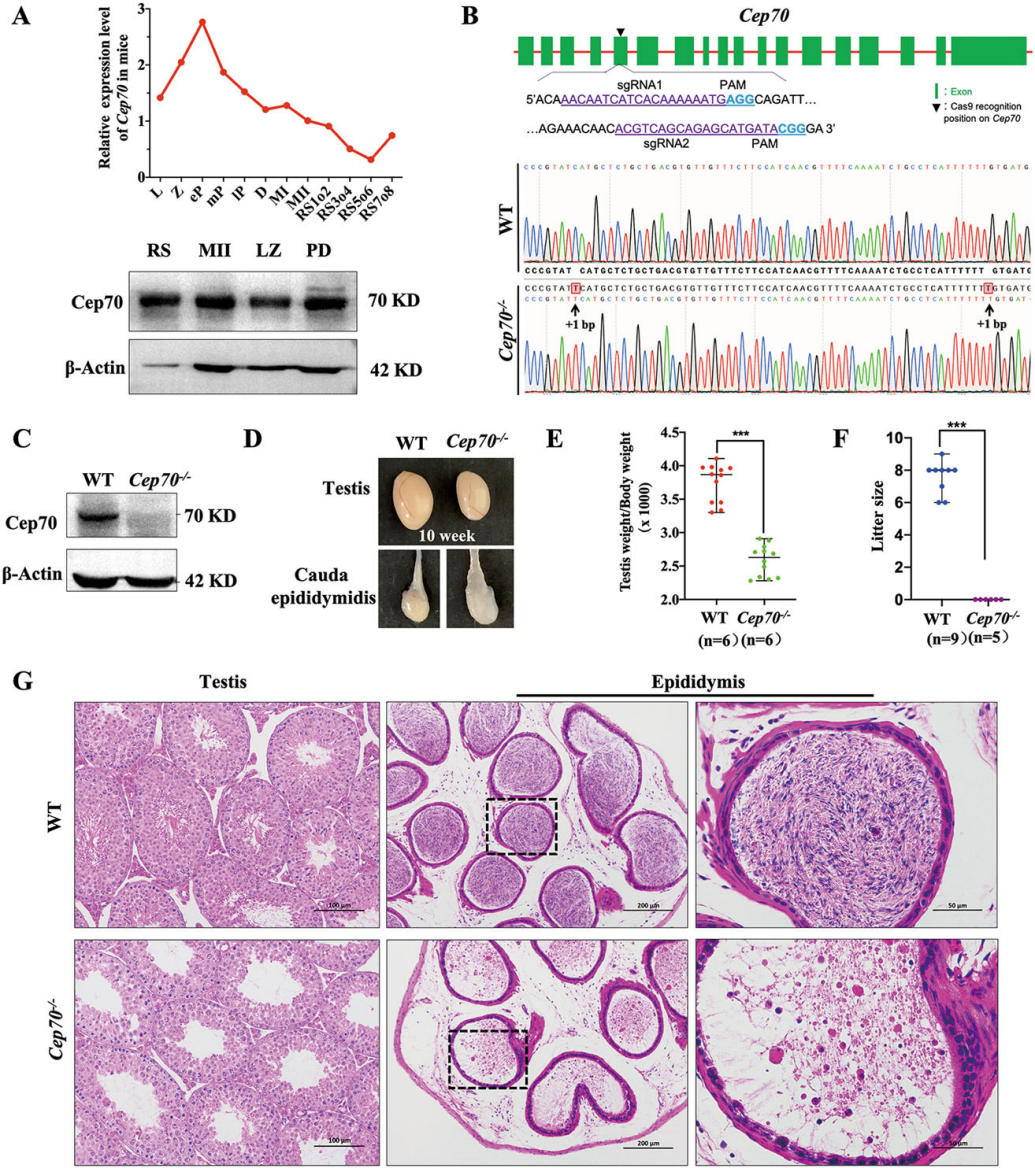
*Cep70* knockout caused male mice infertility. **A** (Up) Relative mRNA levels of *Cep70* in mouse spermatocytes. L: leptotene; Z: zygotene; eP: early pachytene; mP: middle pachytene; lP: late pachytene; D: diplotene; MI: metaphase I; MII: metaphase II; RS1o2: steps 1–2 spermatids; RS3o4: steps 3–4 spermatids; RS5o6: steps 5–6 spermatids; RS7o8: steps 7–8 spermatids. (Down) The expression pattern of protein and mRNA of CEP70 at different staged germ cells isolated by FACS during spermatogenesis in mice. LZ: leptotene and zygotene stage; PD: pachytene and diplotene stage; MII: meiosis II spermatocytes; RS: round spermatids. **B** Schematic diagram of generating *Cep70*-knockout mouse model (*Cep70*^−/−^) by CRISPR/Cas9 system. The genotype analysis results by Sanger sequencing showed a frameshift variant (+1/+1 bp) of *Cep70*^−/−^ mice. **C** Western blotting showed that CEP70 was deleted in 10-week-old *Cep70*^−/−^ whole testis lysates compared to wild type. β-Actin was used as the loading control. **D** Representative images of testis and cauda epididymidis from WT and *Cep70*^−/−^ mice. The testis of *Cep70*^−/−^ was smaller than that of the WT, the cauda epididymis of *Cep70*^−/−^ was more transparent than that of the WT. (**E**) Testis weight to body weight ratio of WT and *Cep70*^−/−^ mice at 10-weeks (*n* = 6). Data are presented as the mean ± SEM. *p* < 0.05 (*), 0.01 (**), or 0.001 (***). **F** Number of pups per litter from male mice (>8-weeks old) naturally crossed with WT female mice (>6-weeks old) for 6 months. *Cep70*^−/−^ male mice showed complete sterility. Data are presented as the mean ± SD, *n* = 5, *p* < 0.001 (***). **G** Histological analysis of the testis and epididymis seminiferous tubules from the WT and *Cep70*^−/−^ mice. No elongated spermatids are present in *Cep70*^−/−^ mice. Scale bar: 100 μm (left); 200 μm (middle); 50 μm (right).

A previous study of single-cell transcriptome data during spermatogenesis showed that *Cep70* mRNA was highly expressed during the spermatogenesis of mice^22^ (Fig. 2A, up). Then, we sorted mouse spermatogenic cells at different stages by FACS and detected CEP70 protein expression in these samples by western blot. The results showed that CEP70 was highly expressed in spermatocytes and round spermatids (Fig. 2A, down).

To study the biological functions of CEP70, we used CRISPR/Cas9 technology to knockout the gene in mice (Fig. 2B). We obtained a mouse strain with two nucleotides inserted in exon five of the *Cep70* gene, which is located downstream of the translation start site. This insertion caused a reading frameshift and created a premature stop codon (Fig. 2B). The deletion of CEP70 was detected by Western blot, and the CEP70 band was only detected in WT testis lysate (Fig. 2C), indicating that the CEP70 protein was successfully depleted in *Cep70*^−/−^ (KO) mice. The growth and healthy of *Cep70*^−/−^ mice were normal, and the *Cep70*^−/−^ females have normal fertility. However, *Cep70*^−/−^ males were completely sterile. Compared to WT male mice, the testis size of *Cep70*^−/−^ male mice was small, and the testes weight was significantly reduced (107.83 ± 5.36 mg versus 68.92 ± 8.45 mg), as shown in Fig. 2D, E. In a 6-months fertility test, the *Cep70*^−/−^ male mice that mated with WT females had no offspring (Fig. 2F). Histological examination showed that the seminiferous tubules of testes and the epididymis of *Cep70*^−/−^ male mice were devoid of elongated spermatids. However, both spermatocytes and round spermatids were present in the *Cep70*^−/−^ male mice testes (Fig. 2G). These results suggest that CEP70 plays an essential role in spermatogenesis.

### CEP70 is dispensable to the prophase of meiosis I during spermatogenesis

**Fig. 3:**
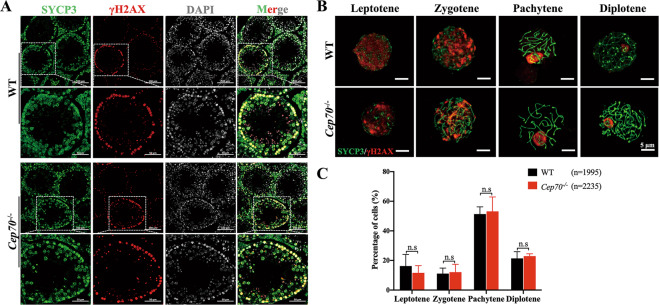
CEP70 is dispensable to the prophase of meiosis I during spermatogenesis. **A** Immunofluorescence staining of SYCP3 (green) and γH2AX (red) in sections of testes from the WT and *Cep70*^−/−^ mice. DAPI indicates the nucleus. Scale bar: 100 μm (up), 50 μm (down). **B** Immunofluorescence staining of SYCP3 (green) and γH2AX (red) in chromosome spreads of spermatocytes from the testes of 10-week-old WT and *Cep70*^−/−^ male mice. Scale bar: 5 μm. **C** Quantification of the proportion of meiotic stages in WT and *Cep70*^−/−^ spermatocytes. The numbers marked in the bars represent the percentage of cells at indicated meiosis stage (leptotene, zygotene, pachytene, and diplotene). For each genotype, three mice were analyzed. *p*-values were calculated by Student’s *t*-test. Data are presented as the mean ± SD, n.s. not statistically significant.

To determine whether CEP70 deletion affects the number of germ cells, including spermatocytes, we performed immunofluorescence staining of MVH, a germ-cell marker, to characterize the first wave of spermatogenesis in mice at 9 days postpartum (dpp), 12 dpp, and 14 dpp that represent the generation time of leptotene, zygotene, and pachytene spermatocytes, respectively^23^. The results indicated that the numbers of germ cells had no significant differences in the first wave of spermatogenesis between WT and *Cep70*^−/−^ male mice, suggesting that the prophase of meiosis I was not affected in *Cep70*-deficient mice (Supplementary Fig. S1A–D). To further confirm this result, we identified different stages of meiotic prophase I by staining of the testes section for phosphorylated H2AX (γH2AX), a marker of unrepaired DNA lesions and the sex body in pachynema, and SYCP3, a component of the synaptonemal complex. The results showed that all stages existed in the spermatocyte nuclei of WT and *Cep70*^−/−^ mice testes (Fig. 3A). To determine whether the ratios of spermatocytes at leptotene, zygotene, pachytene, and diplotene stage were abnormal, we performed spermatocyte spreading of WT and *Cep70*^−/−^ mice testicular tissues (Fig. 3B). The statistical results showed that the lack of CEP70 did not cause abnormal spermatocyte development at all four stages (Fig. 3C). Additionally, we examined the chromosomal synapsis process during the prophase of meiosis I by staining for SYCP3 and SYCP1, important components of the synaptonemal complex. We found that the signals of SYCP1 were similar between WT and *Cep70*^−/−^ mice at pachytene stage (Supplementary Fig. S1E). These results demonstrated that CEP70 deletion had no effects on the prophase of meiosis I.

### CEP70 deletion results in germ-cell apoptosis and abnormal spermiogenesis

**Fig. 4:**
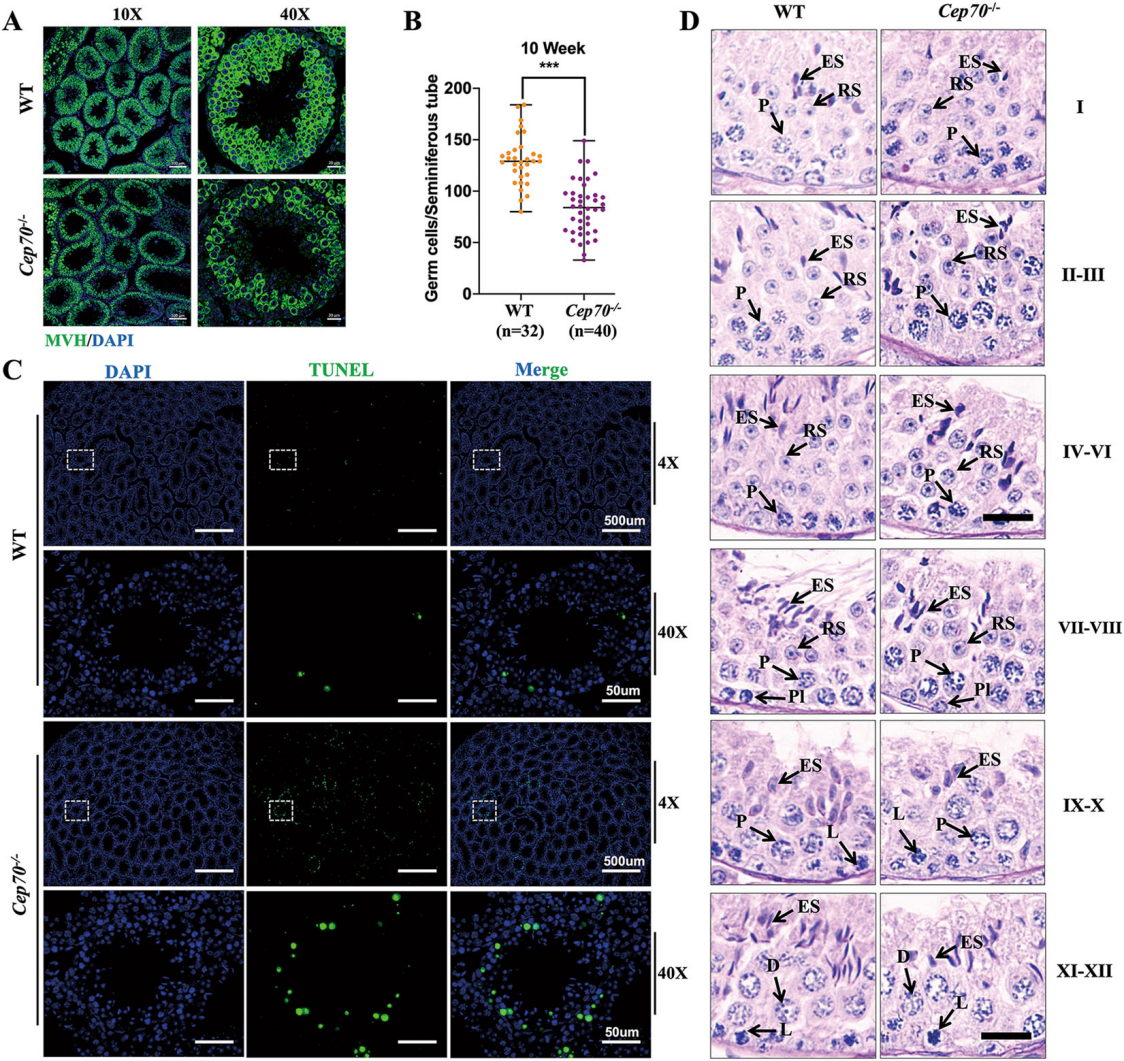
*Cep70-*knockout mice showed germ-cell apoptosis and abnormal spermiogenesis. **A** MVH immunofluorescence staining of testes sections of the WT and *Cep70*^−/−^ mice, demonstrating a significant decrease of germ cells in the seminiferous tubules of *Cep70*^−/−^ mice. Scale bar: 10 μm (left), 20 μm (right). **B** Quantification of germ cells in each seminiferous tubule of the WT (*n* = 32) and *Cep70*^−/−^ mice (*n* = 40) at 10-weeks of age. Data are presented as the mean ± SD, *p* < 0.001 (***). **C** TUNEL immunofluorescence staining of testes sections of the WT and *Cep70*^−/−^ mice. The TUNEL-positive signal (green) level was significantly higher in *Cep70*^−/−^ mice. Scale bar: 500 μm (up), 50 μm (down). **D** PAS-hematoxylin staining of seminiferous tubules from the WT and *Cep70*^−/−^ mice, indicating spermatogenic arrest at stages VII–VIII in the *Cep70*^−/−^ mice. Scale bar: 20 μm.

Although the number of germ cells at 9 dpp, 12 dpp, and 14 dpp of the WT and *Cep70*^−/−^ male mice testis were not affected, the results of MVH staining showed that the loss of CEP70 caused a significant decrease in the number of germ cells at 10-weeks (Fig. 4A, B), probably because the germ cells were undergoing apoptosis. The TUNEL assay results showed that the apoptosis level of germ cells was significantly increased in *Cep70*^−/−^ mice testes (Fig. 4C). To identify exactly which stage of spermatogenesis was affected in *Cep70*-deficient mice, we performed PAS staining to distinguish the 12 stages (I–XII) of spermatogenesis. The results showed that spermatogenesis of *Cep70*-deficient mice was blocked at stages VII and VIII, and most of the round spermatids could not be transformed into elongated spermatids (Fig. 4D). According to previous reports, Sertoli cells play an important role in the stage of spermiogenesis^24^. In order to determine whether *Cep70* deletion affects the development and localization of Sertoli cells, SOX9 (a marker of Sertoli cells) staining results showed that there was no difference in the number and location of Sertoli cells in the testes of WT and *Cep70*^−/−^ male mice (fig. S2). Together, these findings suggest that the absence of CEP70 might lead to abnormal spermiogenesis in male mice and ultimately leads to infertility.

### Loss of CEP70 function results in abnormal formation of flagella and acrosomes

**Fig. 5:**
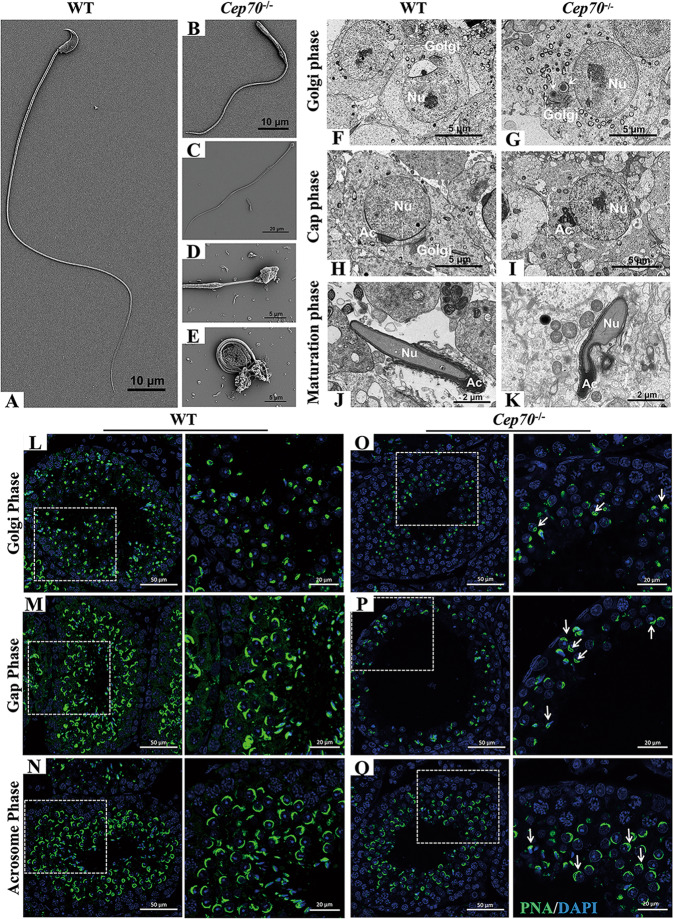
*Cep70*-knockout mice showed the abnormal formation of flagella and acrosomes. **A**–**E** SEM analysis showed the morphology of sperm. Sperm with normal structure (**A**) was observed in the WT mice. Sperm with head defects, including irregular shape (**B**, **D**) and microcephaly (**C**), and tail deformities (**E**) were observed in the *Cep70*^−/−^ mice. **F**–**K** TEM analysis demonstrated the Golgi phase (**F**, **G**), cap phase (**H**, **I**) and maturation phase (**J**, **K**) of the WT (**F**, **H**, **J**) and *Cep70*^−/−^ (**G**, **I**, **K**) mice. Multiple acrosomal structures (**G**), vacuolated acrosomes (**I**), and irregularly shaped acrosomes (**K**) were observed in *Cep70*^−/−^ mice. Nu: nucleus; Ac: acrosome. Scale bar: 5 μm (**F**–**I**), 2 μm (**J**–**K**). **L**–**Q** Immunofluorescence staining with FITC-conjugated PNA (green) on sections of the WT (**L**–**N**), and *Cep70*^−/−^ (**O**–**Q**) mice testes, including Golgi phase (**L**, **O**), gap phase (**M**, **P**), and acrosome phase (**N**, **Q**). Images displayed on the right, show the higher magnification of the boxed areas. Arrows indicate defective acrosome structures. Scale bar: 50 μm (left), 20 μm (right).

To further determine whether there was an abnormality in the process of spermatid head shaping, SEM and TEM were performed. The results of SEM showed that the mature sperm of WT male mice had a complete structure, including obvious equatorial segments, a post-acrosomal sheath, a ventral spur, and a sharp hook rim (Fig. 5A). Although there were spermatozoa with flagella in *Cep70*^−/−^ mice epididymis, the tail and head were abnormal compared to WT mice (Fig. 5B–E). Sperm head defects showed microcephaly and irregular shapes, and tail deformities showed bending, coiling, wrinkling, and shortening. Furthermore, we found that the sperm head of *Cep70*^−/−^ mice also had an abnormal acrosome structure, which suggested that the deletion of CEP70 might also affect the acrosome biogenesis. To verify this hypothesis, we conducted a TEM analysis of the testes. TEM results showed that the Golgi, cap and maturation phases in WT mice testes could be identified by their standard characteristics (Fig. 5F, H, J). However, multiple acrosomal structures could be found in the Golgi phase of *Cep70*^−/−^ mice testes (Fig. 5G). Additionally, the vacuolated or irregularly shaped acrosomes were detected in the cap and maturation phases of *Cep70*^−/−^ mice spermatids (Fig. 5I, K). Moreover, we found that WT and *Cep70*^−/−^ testis sperm flagellar cross-section (principal and end piece) of each structure were not different, according to the TEM analysis (Supplementary Fig. S3). This result demonstrated that the absence of CEP70 did not affect the ultrastructure of sperm flagellar.

To further confirm the defects in acrosome formation in *Cep70*^−/−^ mice, we performed immunofluorescence staining on testis sections with FITC-conjugated peanut agglutinin (PNA), a marker of the outer acrosomal membrane of spermatids. As expected, the WT spermatocytes had normal acrosome at Golgi, cap and acrosome phases (Fig. 5L–N). However, the *Cep70* null spermatocytes exhibited abnormal acrosome structures (e.g., multiple acrosome centers and discrete structures) at all three acrosomal differentiating phases (Fig. 5O–Q), which was similar to observations from TEM analysis. Together, these results indicated that the CEP70 deficient affected the process of acrosome biogenesis. In addition, we found that the total sperm, motile sperm and progressive sperm were significantly decreased by CASA (Supplementary Fig. S4). The data further indicate that CEP70 was involved in acrosome biogenesis and sperm flagellar formation and its loss can lead to OAT-like phenotypes and further cause male infertility.

### CEP70 regulates key proteins of acrosome and flagella formation in spermatogenesis

**Fig. 6:**
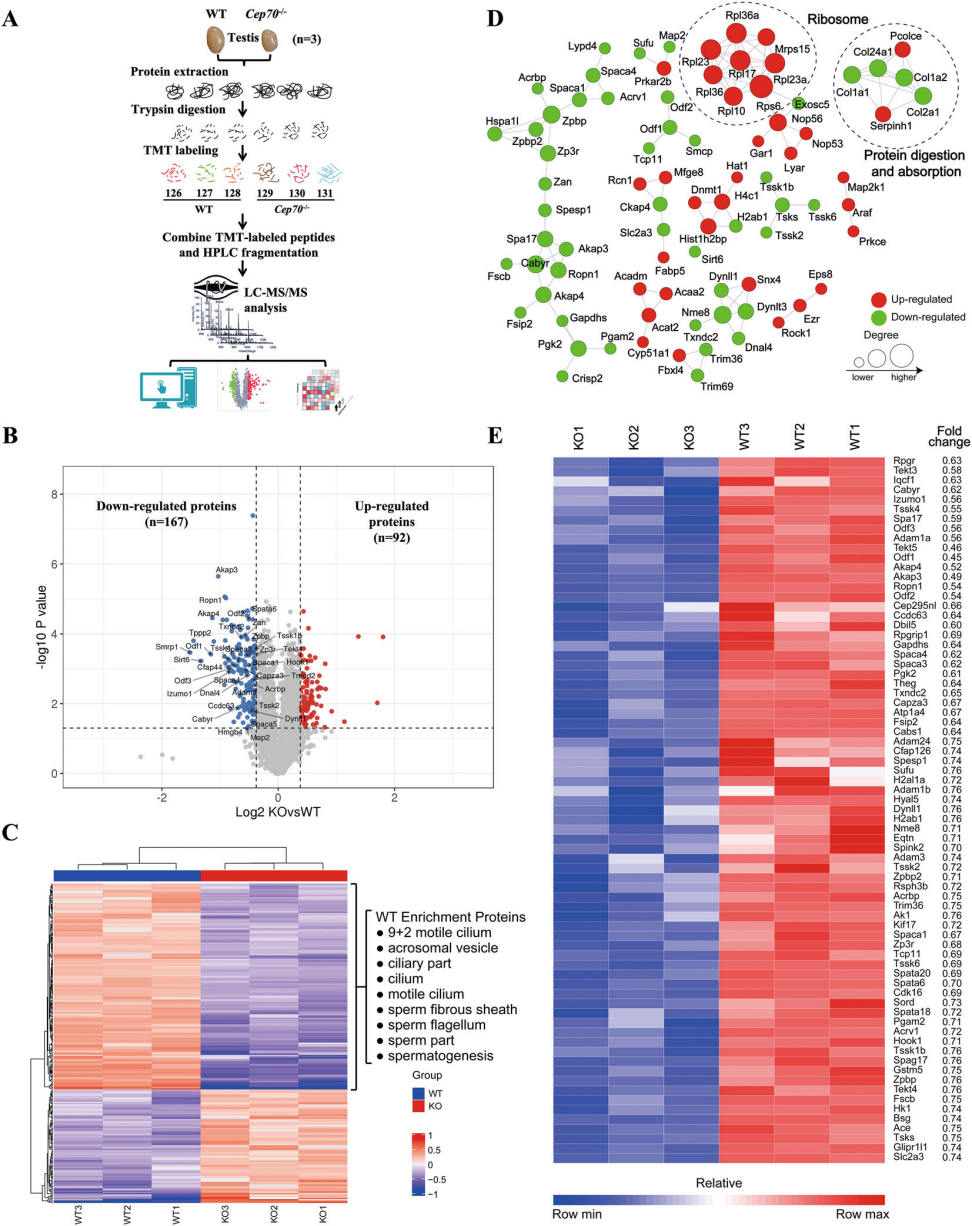
Proteomics analysis verified that CEP70 participated in the regulation of acrosome and flagella formation. **A** Flow diagram of testis TMT-label proteomics analysis of the WT and *Cep70*^−/−^ mice (*n* = 3). **B** Volcano plot show the downregulated (FC < 1/1.3, blue) and upregulated proteins (FC > 1.3, red) in *Cep70*^−/−^ mice testes compared with WT testes. **C** The heatmap of GO terms enriched in DEPs between the WT and *Cep70*^−/−^ mice testes. **D** PPI network analysis revealed the biological processes of the DEPs between the WT and *Cep70*^−/−^ mice testes. **E** Heatmap showing the discrepancy expression level of downregulated proteins between the WT and *Cep70*^−/−^ mice testes.

To investigate the molecular mechanism of CEP70 in the development of acrosome biogenesis and sperm flagellar formation, quantitative proteomic analyses of testes of WT and *Cep70*^−/−^ male mice were performed by TMT labeling, HPLC fractionation and LC–MS/MS analysis (Fig. 6A). The fold change (FC) in the amount of differential expression exceeding 1.3, and less than 1/1.3 (KO/WT) was regarded as the change threshold for a significant upregulation and downregulation, respectively. A total of 4608 proteins were quantified, of which 259 proteins were identified to be differentially expressed (Supplementary Table S3, *p* < 0.05), including 167 downregulated and 92 upregulated proteins (Fig. 6B). GO annotation and enrichment analysis of DEPs in WT and *Cep70*^−/−^ testes showed that 61 DEPs are related to the development of spermiogenesis (e.g., sperm part, acrosomal vesicle, motile cilium, sperm flagellum, 9 + 2 motile cilium, cilium, sperm fibrous sheath, and ciliary part) among the downregulated proteins (Fig. 6C). The results of the PPI network indicated that most downregulated proteins interact with each other and play important functions in spermatogenesis. In addition, the absence of CEP70 also caused the upregulated the expression of proteins related to the biological processes of ribosome and protein digestion and absorption, indicating that these two biological processes may be involved in the regulation of spermatogenesis (Fig. 6D). Furthermore, the heatmap of 73 downregulated proteins displayed sharp differences between WT and *Cep70*^−/−^ male testes and high-quality repeatability between samples in the same group (Fig. 6E).

**Fig. 7:**
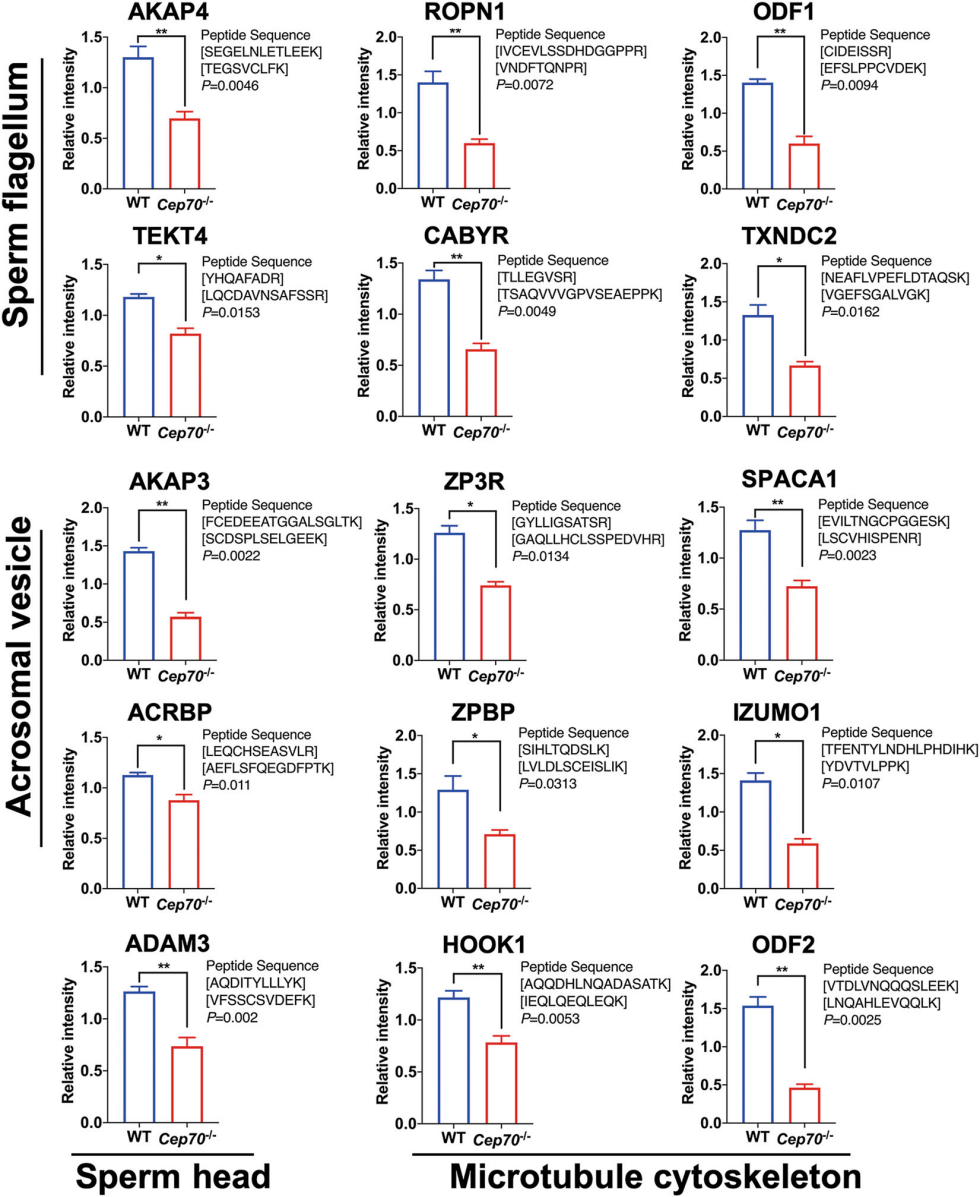
Fifteen proteins were validated in WT and *Cep70*^−/−^ male mice testes by PRM. Fifteen downregulated proteins associated with sperm flagella, sperm head, acrosomal vesicle, and microtubule cytoskeleton, including AKAP4, ROPN1, ODF1, TEKT4, CABYR, TXNDC2, AKAP3, ZP3R, SPACA1, ACRBP, ZBPB, IZUMO1, ADAM3, HOOK1, and ODF2 showed significant differences in *Cep70*^−/−^ male mice testes compared with WT testes (*n* = 3). Data are presented as the mean ± SD. *p* < 0.05 (*), 0.01 (**), or 0.001 (***).

In order to further verify the quality of the proteomic data, we further selected 15 proteins that play important regulatory functions in the process of spermatogenesis from the four terms based on the GO annotation results, including sperm flagellum [A-kinase anchoring protein 4 (AKAP4)^25^, tektin-4 (Tekt4)^26^, outer dense fiber of sperm tails 1 (ODF1)^27^, calcium binding tyrosine phosphorylation regulated (CABYR)^28^, Rhophilin associated tail protein 1 (ROPN1)^29^, and thioredoxin domain-containing protein 2 (TXNDC2)^30,31^], sperm head [A disintegrin and metallopeptidase domain 3 (ADAM3)^32^], acrosomal vesicle [A-kinase anchoring protein 3 (AKAP3)^33^, Zona pellucida sperm-binding protein 3 receptor (ZP3R)^34^, sperm acrosome membrane-associated protein 1 (SPACA1)^35^, Acrosin-binding protein (ACRBP)^36^, Zona pellucida-binding protein (ZPBP)^37,38^, and Izumo sperm-egg fusion protein 1 (IZUMO1)^39,40^], and microtubule cytoskeleton [Protein Hook homolog 1 (HOOK1)^41^, Outer dense fiber of sperm tails 2 (ODF2)^42^], and performed PRM analysis on the testis protein samples of WT and *Cep70*^−/−^ male mice (Supplementary Table S4)^43^. As shown in Fig. 7, the expression levels of all the 15 selected proteins were significantly decreased in *Cep70*^*−/−*^ male mice compared with WT. Moreover, the fold difference of KO/WT ratio was noticeably smaller in PRM than TMT-label quantification. These results indicate that the CEP70 deficiency affects the proteins associated with acrosome and flagella formation during spermatogenesis.

## Discussion

Our previous transcriptomic data demonstrated that *CEP70* is highly expressed during meiosis and spermiogenesis, suggesting that *CEP70* may play an indispensable function during spermatogenesis^13^. The human *CEP70* is located at 3q22.3, with 18 exons that encodes a 597 amino acid protein (~70 kD), and the N terminus (1–326 amino acid) contains two coiled-coil domains, and the C terminus (327–597 amino acid) contains a tetratricopeptide repeat (TPR) domain according to the database of UniProt (https://www.uniprot.org/uniprot/Q8NHQ1#structure). In this study, we investigated the potential role and functional importance of CEP70 in the regulation of acrosome biogenesis and sperm flagellar formation during spermatogenesis by generating *Cep70*^−/−^ mice using CRISPR/Cas9 technology. *Cep70*-deficient male mice show complete infertility and severely impaired spermatogenesis. To the best of our knowledge, this is the first report on the involvement of CEP70 in the regulation of spermatogenesis.

By comparing and analyzing the exon sequences of *CEP70* in azoospermia patients and normal males, we found that four subjects had the same heterozygous mutation site, which was predicted to be a deleterious mutation. Unfortunately, there were no homozygous mutations or compound heterozygous mutations were found in the azoospermia patients, indicating that the recessive CEP70 may just relate to azoospermia and the deleterious heterozygous mutation of CEP70 may increase the risk of azoospermia. Therefore, we hypothesized that CEP70 is involved in the regulation of spermatogenesis, and used CRISPR/Cas9 technology to construct a global knockout model of *Cep70* to study its biological functions. By analyzing the histological changes of the testis and epididymis, we found that CEP70 deficiency resulted in impaired spermatogenesis. Fertility testing revealed that the absence of CEP70 caused complete infertility in male mice.

Then, we found that the loss of CEP70 function caused the spermatogenesis to be blocked at stages VII and VIII, leading to no sperm flagella formation through PAS staining. The concentration of sperm in the epididymis and the number of motile sperm in *Cep70*^−/−^ mice was comparable to that in WT mice by CASA, and there was no motility sperm in the epididymis of *Cep70*^−/−^ mice. The main manifestation of oligospermia is a significant decrease in the number of spermatids due to the apoptosis of germ cells or the failure of sperm release from the seminiferous epithelium^44^. The TUNEL assay showed that germ cells had obvious apoptosis in *Cep70*^−/−^ mice. By comparing the testicular proteome data of WT and *Cep70*^−/−^ mice, the loss of CEP70 caused a significant downregulation of the expression of DYNLL1, leading to the high expression of pro-apoptotic proteins that cause germ-cell apoptosis^45,46^. More remarkably, through SEM analysis we found that sperm lacking CEP70 had abnormal acrosomes and irregular tails; thus, the poor progressive motility in *Cep70*^−/−^ spermatozoa could be attributed to the abnormal head and flagellar morphology. An earlier study showed that the zebrafish homolog of CEP70 can promote cilia assembly by controlling the length of the axoneme in zebrafish embryos^21^. Through this series of phenotypic analyses, we determined that the loss of CEP70 caused the OAT phenotype (low spermatozoa count, poor sperm motility, and abnormal sperm head structure) of male mice^47,48^. OAT, partially caused by defects in spermiogenesis, is the most common clinical profile in infertile men, including oligozoospermia, asthenozoospermia, and teratozoospermia^49,50^. However, non-obstructive azoospermia (NOA) is caused by a critical failure in the early stage of spermatogenesis (abnormal sperm self-renewal or meiotic arrest), which is different from OAT symptom^49^. Overall, we determined that CEP70 participates in the regulation of spermiogenesis, and the genetic alteration of *Cep70* might related to OAT phenotype. According to the results of TEM and PNA immunofluorescence staining, CEP70 plays an important role in acrosome formation. Analysis of the proteomic data revealed that the components of the acrosome matrix (ZP3R and ZAN) in *Cep70*^−/−^ mice were significantly downregulated compared to WT mice^51^. After capacitation, ZP3R (also named SP56), a zona pellucida-binding protein, is located on the sperm surface and released at the time of the fusion of outer acrosomal and plasma membranes^52^. Additionally, the expression of another two membrane proteins (IZUMO1 and SPACA1) required for head shaping and oocyte fusion in *Cep70*^−/−^ mice testes were significantly decreased compared to WT testes^53,54^. Therefore, CEP70 plays an important regulatory function in the process of acrosome formation.

Additionally, 61 downregulated proteins associated with the formation of sperm tail according to GO annotation were identified in the *Cep70*^−/−^ mice compared with the WT mice. Among them, it has been reported that the deletion of some genes (*Spata6*, *Ropn1*, and *Cabyr*) in a mouse model showed abnormalities and deformities of the sperm tail structure^55^. Studies have shown that the ROPN1 and CABYR expression level decreased remarkably in asthenospermia, and they can bind to AKAP3 to regulate the PKA signaling pathway and affect sperm flagella formation^28,56^. The depletion of spermatogenesis associated 6 (SPATA6) disrupts the proper formation of the sperm connecting piece and head-tail conjunction^57^. Furthermore, testis-specific serine/threonine-protein kinase 1, 2, and 4 (TSSK1, 2, and 4) regulate the development of sperm flagellum^58,59^. In our analysis of the proteomics data, it was found that the expression of ROPN1, CABYR, SPATA6, TSSK1, TSSK2, TSSK4, and AKAP3 decreased significantly after CEP70 deficiency, so the sperm of *Cep70*^−/−^ mice showed a phenotype of flagella formation failure. Moreover, the mutation of CAPZA3 can cause male infertility and show an OAT-like phenotype, which may also be one of the important reasons for the OAT phenotype caused by CEP70 deletion^60^.

In summary, our genetic and functional data, based on human subjects and mouse models, strongly suggest that heterozygous deleterious mutations of CEP70 may be a novel genetic cause of OAT. Additionally, the underlying mechanism that CEP70 regulates sperm flagellar development was elucidated in this study. The screening of the deleterious mutations of *CEP70* could be important for clinical molecular diagnosis of male infertility. As the findings about CEP70 in this study are novel, further research is required on this unfamiliar gene to explore the importance of *CEP70* in male fertility.

## Materials and methods

### Mutation screening and sanger sequencing

To scan for *CEP70* mutations in infertile male patients, we used DNA samples collected from 476 infertile patients with azoospermia and 252 men with normal fertility, from the our reproductive Center between 2013 and 2016 to perform *CEP70* exon amplification. The collection of these samples was approved in 2012 by the Reproductive Medicine Ethics Committee of Peking University Third Hospital (Item number: 2012sz018). The OneTaq® Hot Start Quick-Load® 2X Master Mix (New England Biolabs, M0488S) was used for PCR amplification, and the 14 coding exon sequences of CEP70 were analyzed by Sanger sequencing using an ABI 3730xl DNA sequencer (Applied Biosystems™) by BeiJing Ruibio Biotech Co., Ltd. The sequencing results were compared with the reference genome sequence of CEP70. The primers used for the PCR are listed in Supplementary Table S2.

### Generation of the *Cep70*-knockout mouse model

According to experimental procedures similar to those reported previously^61,62^, a *Cep70* (NCBI: NM_023873.4) knockout mouse model (termed *Cep70*^*−/−*^) with the frameshift variant was obtained using the CRISPR/Cas9 system, as shown in Fig. 1A. Two pairs of single-guide RNAs (sgRNAs) were designed for *Cep70* on exon five. Sanger sequencing was used to identify the genotype of the founder mouse and its offspring with a frameshift variant. The sequence information of the sgRNAs and genotyping primers are listed in Supplementary Table S1. Mice, with a C57BL/6J genetic background, were maintained under controlled temperature (20–22 °C), appropriate humidity (50–70%), lighting conditions (12/12 h light/dark cycle), and food and water provided ad libitum. The *Cep70*-knockout mice used in the experiment were 8-weeks of age or older. All experimental procedures and animal care were approved by the Animal Care and Use Committee of the Peking University Health Science Center. During the experiment, the mice in the WT group and the KO group were randomly selected without any subjective factors, and the number of animal samples in each experiment was guaranteed to be at least 3 or more.

### Fertility assessment of mice

To assess the fertility of the *Cep70*^−/−^ mice, sexually mature male (>8-weeks old, *n* = 5) and female (>6-weeks old, *n* = 5) *Cep70*^−/−^ mice were mated with two wild-type (WT) female (>6-weeks old) and male (>8-weeks old) mice for 6 months. During this period, the two WT female mice were replaced every other gestation cycle. Meanwhile, WT female (>6-weeks old) and male mice (>8-weeks old) were mated 1:2, as the control group (*n* = 5). Litter sizes were recorded during the fertility assessment of *Cep70*^−/−^ and WT mice.

### Histological and immunofluorescence analyses

For histological examination, testes and epididymis were obtained from 10-week old *Cep70*^−/−^ and WT males, immediately fixed in modified Bouin’s fixative or 4% paraformaldehyde (PFA) for 24 h and stored in 75% ethanol. Periodic acid-Schiff (PAS) and hematoxylin-eosin (H&E) staining were performed on formalin-fixed, paraffin-embedded testis, and epididymis sections (3–4 μm). The PAS dye solution set was used for PAS staining (Servicebio, G1008), according to manufacturer instructions.

For immunofluorescence analysis, the slices were deparaffinized with xylene and alcohol, retrieved in ethylenediaminetetraacetic acid (EDTA) antigen retrieval buffer (pH 8.0 or 9.0) and placed at room temperature to cool down. Then, the tissue sections were blocked with 3% bovine serum albumin (BSA) for 1 h, and incubated overnight at 4 °C with the following antibodies: anti-MVH (Abcam, ab13840, 1:500, rabbit), anti-SYCP3 (Abcam, ab97672, 1:100, mouse), anti-γH2AX (Abcam, ab11174, 1:400, rabbit), anti-SOX9 (Novus Biologicals, NBP1-85551, 1:100, rabbit), and lectin PNA with Alexa Fluor™ 488 Conjugate (ThermoFisher, L21409, 1:200). The slides were then washed three times with phosphate-buffered saline (PBS) and incubated for 1 h with goat anti-rabbit Alexa Fluor™ 488- or 594-conjugated secondary antibody (Invitrogen, A32766). After washing three times with PBS, the slides were mounted with ProLong™ Gold Antifade Mountant with 4ʹ,6-diamidino-2-phenylindole (DAPI) (Invitrogen, P36931) and then imaged using a Zeiss LSM880 confocal microscope.

### Meiotic nuclear spreading and immunofluorescence staining

Testes were collected from adult male mice, and spermatocyte surface spreading were made as previously reported^63^. In brief, seminiferous tubules were treated with hypotonic extraction buffer (HEB, pH 8.2) for 30–60 min, and then pulverized in 100 mM sucrose buffer (pH 8.2) and gently pipetted to form a cell suspension. The suspension was loaded onto adhesive slides containing 1% PFA and 0.15% Triton X-100 fixative (pH 9.2). After 2 h of incubation, the slides were air-dried overnight. Finally, the slides were washed with PBS (pH 7.4) three times (5 min each) and immunostained with primary antibodies, including anti-SYCP3 (a gift from Hengyu Fan lab, 1:400, rat), anti-SYCP1 (Abcam, ab15090, 1:200, rabbit), and anti-γH2AX (Abcam, ab11174, 1:400, rabbit), according to the above immunofluorescence protocol.

### Isolation of spermatogenic cells

To isolate different types of spermatogenic cells (LZ: leptotene/zygotene spermatocytes; PD: pachytene/diplotene spermatocytes; MII: meiosis II spermatocytes; and RS: round spermatids), we performed fluorescence-activated cell sorting (FACS) using a Flow Cytometer ARIA II (BD Biosciences), as previously reported^22^. Briefly, testes of 10-week-old WT and *Cep70*^−/−^ males mice were incubated in 5 mL Hank’s Balanced Salt Solution (HBSS) with collagenase type I (100 U/mL, Gibco, 17100-017) after removal of the tunica albuginea at 34 °C for 10 min. The digested testes were further digested with 5 mL trypsin (0.25%) containing 100 μL DNase I (5 mg/mL) at 34 °C for 8 min, and then terminated by adding 500 μL fetal bovine serum (FBS). The cell suspension was filtered through a 70 μm cellular filter and centrifuged, then resuspended in Dulbecco’s Modified Eagle Medium (DMEM) with Hoechst 33342 (Invitrogen, H3570, 10 mg/mL) and incubated at 34 °C for 30 min at low-speed rotation. Before sorting, the digested cells were stained with propidium iodide (PI, 1 μg/μL). Then, the digested cells were filtered through a 40 μm cellular filter and sorted by FACS, based on Hoechst 33342/PI staining, into two distinct channels.

### Western blot analyses

To detect the CEP70 protein, testes of *Cep70*^−/−^ and WT male mice were pulverized in liquid nitrogen and collected in 500 μL ice-cold Radio Immunoprecipitation Assay Lysis Buffer (RIPA) buffer containing protease inhibitors. After that, the lysates were diluted with 2× Laemmli sample buffer (Bio-Rad, 1610737), and boiled in water for 10 min. The protein samples were separated by Sodium Dodecyl Sulfate-Polyacrylamide Gel Electrophoresis (SDS-PAGE, 10% acrylamide running gel) and then electrically transferred to polyvinylidene fluoride (PVDF) membranes. After transfer, the PVDF membranes were blocked with 5% skimmed milk in Tris-buffered saline (10 mM Tris, 150 mM NaCl, pH 7.5) containing 0.1% Tween-20 (TBST) for 1 h at room temperature and then immunoblotted at 4 °C overnight with Anti-CEP70 antibody (Abcam, ab227456, 1:1000, Rabbit) and Anti-β-actin mouse monoclonal antibody (TransGen Biotech, HC201, 1:1000). After washing in TBST, the membranes were incubated for 1 h with Anti-rabbit or mouse HRP-conjugated secondary antibody (1:3000). Finally, protein bands were visualized by an enhanced chemiluminescence detection system (Tanon-5200). Western blot images were processed using ImageJ software (Wayne Rasband, USA).

### Sperm motility assays

Sperm motility measurements were performed as previously described^64^. Briefly, the cauda epididymis was dissected from an adult mouse and cut into pieces in 1 mL of human tubal fluid (HTF), and exuded using the “swim-out” method at 37 °C for 30 min to collect sperm. Then, 10 μL of the exudate was put into a glass cell chamber (Leja Products BV, Nieuw-Vennep, The Netherlands) and kept on a heating platform at 37 °C. The spermatozoa was further observed through a ×20 objective lens (Olympus BX51 microscope). A CCD camera (Olympus) was used to image the observation area of each chamber. Spermatozoa samples were analyzed using computer-aided sperm analysis (CASA, CEROS v.12, Hamilton Thorne Research), which were implemented using Minitube sperm visual digital semen assessment system (12500/1300, Minitube Group, Tiefenbach, Germany). The total number of sperm, motile sperm and the proportion of progressive sperm were analyzed.

### Scanning and transmission electron microscopy

For scanning electron microscopy (SEM), epididymal sperm of WT and *Cep70*^−/−^ male mice (10-weeks old) were collected and shredded in HTF medium using the “swim-out” method at 37 °C, shaken every 10 min three times. Then, the supernatant containing sperm was placed in a clean centrifuge tube and pelleted at 300 × *g* for 5 min. After that, the sperm was resuspended in an electron microscopy fixative (Servicebio, G1102), fixed for 2 h at room temperature, and transferred to 4 °C for preservation. Sperm samples were blocked with 1% OsO4 in 0.1 M PBS (pH = 7.4) for 1–2 h at room temperature. After washing in PBS three times (15 min each), the sperm samples were gradually dehydrated in increasing concentrations of ethanol (30%, 50%, 70%, 80%, 90%, 95%, and 100% twice; 15 min each) and in isoamyl acetate (Sinaopharm Group Chemical Reagent Company, 10003128) for 15 min. After that, the samples were dried with a critical point dryer (Quorum, K850). Specimens were attached to metallic stubs using carbon stickers and sputter-coated with gold for 30 s using a Lon sputtering apparatus (Hitachi, MC1000). Finally, the samples were imaged using a scanning electron microscope (Hitachi, SU8100) by the Wuhan Servicebio Technology Company.

For transmission electron microscopy (TEM), the size of testes should be no more than 1 mm^3^. The testes samples were fixed in an electron microscopy fixative at room temperature for 1 h and then transferred into an Eppendorf (EP) tube with fresh TEM fixative for further fixation. Then, the tissues were washed with 0.1 M PBS (pH 7.4) three times for 15 min each. The tissues were fixed with 1% OsO4 in PBS for 2 h at room temperature, and then rinsed in PBS three times for 15 min each. The samples were gradually dehydrated in increasing concentrations of ethanol (30%, 50%, 70%, 80%, 95%, and 100% twice; 20 min each) and in 2 Â acetone (Sinaopharm Group Chemical Reagent Co. LTD, 10000418) for 15 min. The samples were then infiltrated and embedded in resin according to the following steps: acetone: EMBed 812 (SPI, 90529-77-4) = 1:1 for 2–4 h at 37 °C; acetone: EMBed 812 = 1:2 overnight at 37 °C; and pure EMBed 812 for 5–8 h at 37 °C. After that, pure EMBed 812 was poured into the embedding model, the tissues were inserted into the pure EMBed 812, and then kept at 37 °C overnight. The embedding models with resin and samples were moved into a 65 °C oven to polymerize for more than 48 h. Then, the resin blocks were removed from the embedding models for standby application at room temperature. The resin blocks were cut to 60–80 nm thickness using an ultra-microtome (Leica, Leica UC7), and the tissues were fished onto 150 mesh cuprum grids with formvar film. The copper mesh was stained in the dark with 2% uranium acetate saturated alcohol solution for 8 min, rinsed in 70% ethanol three times, and then rinsed in ultrapure water three times, 2.6% lead citrate was used to avoid CO2 staining for 8 min, and then rinsed with ultrapure water three times. After drying with filter paper, the cuprum grids were placed into the grid board and dried overnight at room temperature. The cuprum grids were observed under a transmission electron microscope (Hitachi, HT7800) and images were taken by the Wuhan Servicebio Technology Company.

### TUNEL assays

Terminal deoxynucleotidyl transferase dUTP nick end labeling (TUNEL) assay was carried out according to the manufacturer instructions of In Situ Cell Death Detection Kit (Roche, 11684817910). Images were captured using a laser scanning confocal microscope (3D HISTECH, Pannoramic MIDI).

### Proteomics analysis of testes of WT and *Cep70*^−/−^ male mice

After protein extraction and trypsin digestion of testis of WT (*n* = 3) and *Cep70*^−/−^ (*n* = 3) male mice, the peptide was desalted by Strata X C18 SPE column (Phenomenex) and vacuum-dried. The peptide was reconstituted in 0.5 M TEAB and processed according to the manufacturer protocol for tandem mass tag (TMT) kit/isobaric tags for relative and absolute quantitation (iTRAQ) kit. Briefly, one unit of TMT/iTRAQ reagent were thawed and reconstituted in acetonitrile. The peptide mixtures were then incubated for 2 h at room temperature and pooled, desalted, and dried by vacuum centrifugation. After TMT/iTRAQ labeling, the tryptic peptides were fractionated into fractions by high pH reverse-phase high-performance liquid chromatography (HPLC) using Thermo Betasil C18 column (5 μm particles, 10 mm ID, 250 mm length). After HPLC fractionation, the tryptic peptides were analyzed by LC-tandem mass spectrometry (MS/MS). The resulting MS/MS data were processed using Maxquant search engine (v.1.5.2.8). False discovery rate (FDR) was adjusted to <1% and minimum score for modified peptides was set >40.

According to the data quality control and search, a Gene Ontology (GO) annotation proteome was derived from the UniProt-GOA database (http://www.ebi.ac.uk/GOA/). Then proteins were classified by GO annotation based on three categories: biological process, cellular component and molecular function. Identified proteins domain functional description were annotated by InterProScan (a sequence analysis application, http://www.ebi.ac.uk/interpro/) based on protein sequence alignment method, and the InterPro domain database was used. The Kyoto Encyclopedia of Genes and Genomes (KEGG) database was used to annotate protein pathway. For functional enrichment of GO, pathway, and protein domain, a two-tailed Fisher’s exact test of was employed to test the enrichment of the differentially expressed proteins (DEPs) against all identified proteins based on GO annotation, the KEGG database, and the Interpro domain database. A corrected *p*-value < 0.05 was considered as significant.

Further hierarchical clustering was based on DEP functional classifications (such as GO, domain, pathway, and complex). We first collated all the categories obtained after enrichment along with their *p*-values, and then filtered for those categories which were at least enriched in one of the clusters with a *p*-value < 0.05. This filtered *p*-value matrix was transformed by the function *x* = −log10 (*p*-value). Finally, the *x* values were *z*-transformed for each functional category. These *z*-scores were then clustered by one-way hierarchical clustering (Euclidean distance and average linkage clustering) in Genesis. Cluster membership was visualized by a heatmap using the “heatmap.2” function from the “gplots” R-package.

Protein–protein interaction (PPI) networks of all the DEPs, database accession, or sequence, were searched against the STRING database (version 10.1). Only interactions between the proteins belonging to the searched data set were selected, thereby excluding external candidates. STRING defines a metric called “confidence score” to define interaction confidence; we fetched all interactions that had a confidence score ≥ 0.7 (high confidence). The interaction network from STRING was visualized in the R-package “networkD3.” The TMT proteomics analysis in our research was supported by Jingjie PTM BioLabs.

### Parallel reaction monitoring (PRM)

The trypsin-digested peptides of WT and *Cep70*^−/−^ male mice testes were dissolved in an aqueous solution containing 0.1% formic acid and 2% acetonitrile and then subjected to gradient treatment with an aqueous solution containing 0.1% formic acid and 90% acetonitrile, all at a constant flow rate of 500 nL/min on an EASY-nLC 1000 ultra-performance liquid chromatography (UPLC) system (ThermoFisher Scientific). After treatment, the peptides were subjected to a Nanospray ionization (NSI) source followed by MS/MS in a Q ExactiveTM Plus (ThermoFisher Scientific) coupled online to the UPLC. The ion source voltage was set to 2.1 kV, and the peptide precursor ions and their secondary fragments were detected and analyzed by high-resolution Orbitrap. The *m*/*z* scan range was 350–1000 for the full MS at a resolution of ×70,000, and peptides were then selected for MS/MS using normalized collision energy (NCE) setting of 27, and the fragments were detected in the Orbitrap at a resolution of ×17,500. The data acquisition mode uses the data-independent acquisition (DIA) program that alternated between one MS scan followed by 20 PRM scans, and the fragmentation energy of the higher energy collisional dissociation (HCD) was set to 27. The target of automatic gain control (AGC) and maximum injection time (Max IT) for full MS and MS/MS were set at 3E6/50 ms and 1E5/200 ms, respectively. The isolation window for MS/MS was set at 1.6 *m*/*z*. PRM data were manually curated within Skyline (version 3.6)^65^. The peptide parameters were set as follows: enzyme was set as Trypsin [KR/P], and max missed cleavage was set as 2 and the peptide length was set as 7–25 amino acid residues, and variable modification was set as alkylation on cysteine. The transition parameters were set as follows: precursor charges were set as 2, 3, ion charges were set as 1, ion types were set as b, y. The product ions were set from ion 3 to last ion, the ion match tolerance was set as 0.02 Da.

### Statistical analysis

Data are presented as the mean ± standard deviation (SD) of at least three independent replicates, and error bars indicate SD. Statistical analyses were performed using a two-tailed Student’s *t*-test (*n* ≥ 3). Differences were considered significant at *p* < 0.05 (*), *p* < 0.01 (**), and *p* < 0.001 (***).

## Supplementary information

Table S3 The detail information of different expression proteins between WT and Cep70 KO male mice testes

Table S4 The relative abundance data of phosphopeptides from WT and Cep70 KO male mice testis lysates

Supplementary Materials

## Data Availability

All data, including its supplementary information files, supporting the findings of this study are included in this published article. The mass spectrometry proteomics data have been deposited to the ProteomeXchange Consortium (http://proteomecentral.proteomexchange.org) via the iProX partner repository with the data set identifier PXD023680.

## References

[1] Luk BH, Loke AY (2016). A review of supportive interventions targeting individuals or couples undergoing infertility treatment: directions for the development of interventions. J. Sex Marital Ther..

[2] Esteves SC, Chan P (2015). A systematic review of recent clinical practice guidelines and best practice statements for the evaluation of the infertile male. Int. Urol. Nephrol..

[3] Babakhanzadeh E, Nazari M, Ghasemifar S, Khodadadian A (2020). Some of the factors involved in male infertility: a prospective review. Int. J. Gen. Med..

[4] Cannarella R, Condorelli RA, Mongioì LM, La Vignera S, Calogero AE (2020). Molecular biology of spermatogenesis: novel targets of apparently idiopathic male infertility. Int. J. Mol. Sci..

[5] Tuttelmann F, Ruckert C, Ropke A (2018). Disorders of spermatogenesis: perspectives for novel genetic diagnostics after 20 years of unchanged routine. Med. Genet..

[6] Matzuk MM, Lamb DJ (2008). The biology of infertility: research advances and clinical challenges. Nat. Med..

[7] Kanatsu-Shinohara M, Shinohara T (2013). Spermatogonial stem cell self-renewal and development. Annu. Rev. Cell Dev. Biol..

[8] Xu Z (2020). CIB4 is essential for the haploid phase of spermatogenesis in mice†. Biol. Reprod..

[9] Oakberg EF (1956). Duration of spermatogenesis in the mouse and timing of stages of the cycle of the seminiferous epithelium. Am. J. Anat..

[10] Hermo L, Pelletier RM, Cyr DG, Smith CE (2010). Surfing the wave, cycle, life history, and genes/proteins expressed by testicular germ cells. Part 2: Changes in spermatid organelles associated with development of spermatozoa. Microsc. Res. Tech..

[11] de Kretser DM, Loveland KL, Meinhardt A, Simorangkir D, Wreford N (1998). Spermatogenesis. Hum. Reprod..

[12] Jan SZ (2017). Unraveling transcriptome dynamics in human spermatogenesis. Development.

[13] Wang M (2018). Single-cell RNA sequencing analysis reveals sequential cell fate transition during human spermatogenesis. Cell Stem Cell.

[14] Amaral A (2013). Human sperm tail proteome suggests new endogenous metabolic pathways. Mol. Cell Proteom..

[15] Andersen JS (2003). Proteomic characterization of the human centrosome by protein correlation profiling. Nature.

[16] Shi X (2011). CEP70 Protein interacts with ?-tubulin to localize at the centrosome and is critical for mitotic spindle assembly. J. Biol. Chem..

[17] Sha YW (2017). A homozygous CEP135 mutation is associated with multiple morphological abnormalities of the sperm flagella (MMAF). Gene.

[18] Hall EA (2013). Acute versus chronic loss of mammalian Azi1/Cep131 results in distinct ciliary phenotypes. PLoS Genet..

[19] Shi X (2012). Cep70 promotes microtubule assembly in vitro by increasing microtubule elongation. Acta Biochim. Biophys. Sin..

[20] Shi X (2015). Cep70 regulates microtubule stability by interacting with HDAC6. FEBS Lett..

[21] Wilkinson CJ, Carl M, Harris WA (2009). Cep70 and Cep131 contribute to ciliogenesis in zebrafish embryos. BMC Cell Biol..

[22] Chen Y (2018). Single-cell RNA-seq uncovers dynamic processes and critical regulators in mouse spermatogenesis. Cell Res..

[23] Lei WL, Han F, Hu MW, Liang QX, Sun QY (2020). Protein phosphatase 6 is a key factor regulating spermatogenesis. Cell Death Differ.

[24] Wu S, Yan M, Ge R, Cheng CY (2020). Crosstalk between sertoli and germ cells in male fertility. Trends Mol. Med..

[25] Fang X (2019). Proteomics and single-cell RNA analysis of Akap4-knockout mice model confirm indispensable role of Akap4 in spermatogenesis. Dev. Biol..

[26] Roy A, Lin YN, Agno JE, Demayo FJ, Matzuk MM (2007). Absence of tektin 4 causes asthenozoospermia and subfertility in male mice. FASEB J..

[27] Yang K, Grzmil P, Meinhardt A, Hoyer-Fender S (2014). Haplo-deficiency of ODF1/HSPB10 in mouse sperm causes relaxation of head-to-tail linkage. Reproduction.

[28] Li YF (2011). CABYR binds to AKAP3 and Ropporin in the human sperm fibrous sheath. Asian J. Androl..

[29] Fiedler SE, Dudiki T, Vijayaraghavan S, Carr DW (2013). Loss of R2D2 proteins ROPN1 and ROPN1L causes defects in murine sperm motility, phosphorylation, and fibrous sheath integrity. Biol. Reprod..

[30] Yang Y, Richard O, Antonio MV (2002). Developmental expression of spermatid-specific thioredoxin-1 protein: transient association to the longitudinal columns of the fibrous sheath during sperm tail formation. Biol. Reprod..

[31] Smith TB, Baker MA, Connaughton HS, Habenicht U, Aitken RJ (2013). Functional deletion of Txndc2 and Txndc3 increases the susceptibility of spermatozoa to age-related oxidative stress. Free Radic. Biol. Med..

[32] Fujihara Y (2019). Identification of multiple male reproductive tract-specific proteins that regulate sperm migration through the oviduct in mice. Proc. Natl Acad. Sci. USA.

[33] Xu, K., Yang, L., Zhang, L. & Qi, H. Lack of AKAP3 disrupts integrity of the subcellular structure and proteome of mouse sperm and causes male sterility. *Development* **147**, dev181057 (2020). 10.1242/dev.181057 31969357

[34] Buffone MG (2008). Recombinant mouse sperm ZP3-binding protein (ZP3R/sp56) forms a high order oligomer that binds eggs and inhibits mouse fertilization in vitro. J. Biol. Chem..

[35] Jiang S (2019). Fluoride exposure arrests the acrosome formation during spermatogenesis via down-regulated Zpbp1, Spaca1 and Dpy19l2 expression in rat testes. Chemosphere.

[36] Yoshinori K (2016). Biogenesis of sperm acrosome is regulated by pre-mRNA alternative splicing of Acrbp in the mouse. Proc. Natl Acad. Sci. USA.

[37] Jiang S (2019). Fluoride exposure arrests the acrosome formation during spermatogenesis via down-regulated Zpbp1, Spaca1 and Dpy19l2 expression in rat testes. Chemosphere.

[38] Chen SR (2016). The control of male fertility by spermatid-specific factors: searching for contraceptive targets from spermatozoon’s head to tail. Cell Death Dis..

[39] Satouh Y, Inoue N, Ikawa M, Okabe M (2012). Visualization of the moment of mouse sperm-egg fusion and dynamic localization of IZUMO1. J. Cell Sci..

[40] Zhou C, Huang L, Shi DS, Jiang JR (2017). Effects of latrunculin A on the relocation of sperm IZUMO1 during gamete interaction in mouse. Mol. Reprod. Dev..

[41] Mendoza-Lujambio I (2002). The Hook1 gene is non-functional in the abnormal spermatozoon head shape (azh) mutant mouse. Hum. Mol. Genet..

[42] Donkor FF (2004). Outer dense fibre protein 2 (ODF2) is a self-interacting centrosomal protein with affinity for microtubules. J. Cell Sci..

[43] Bourmaud A, Gallien S, Domon B (2016). Parallel reaction monitoring using quadrupole-orbitrap mass spectrometer: principle and applications. Proteomics.

[44] O’Donnell L, Nicholls PK, O’Bryan MK, McLachlan RI, Stanton PG (2011). Spermiation: the process of sperm release. Spermatogenesis.

[45] Jurado S (2012). The Zinc-finger protein ASCIZ regulates B cell development via DYNLL1 and Bim. J. Exp. Med..

[46] King A (2017). Dynein light chain regulates adaptive and innate B cell development by distinctive genetic mechanisms. PLoS Genet..

[47] Jungwirth A (2012). European Association of Urology guidelines on male infertility: the 2012 update. Eur. Urol..

[48] Sha YW (2018). TDRD6 is associated with oligoasthenoteratozoospermia by sequencing the patient from a consanguineous family. Gene.

[49] O’Donnell L, McLachlan RI, Merriner DJ, O’Bryan MK, Jamsai D (2014). KATNB1 in the human testis and its genetic variants in fertile and oligoasthenoteratozoospermic infertile men. Andrology.

[50] Nasirshalal M (2020). Identification of the PRM1 gene mutations in oligoasthenoteratozoospermic men. Andrologia.

[51] Crapster JA, Rack PG, Hellmann ZJ, Le AD, Chen JK (2020). HIPK4 is essential for murine spermiogenesis. eLife.

[52] Kim K-S (2001). Mouse sperm protein sp56 is a component of the acrosomal matrix. Biol. Reprod..

[53] Inoue N, Ikawa M, Isotani A, Okabe M (2005). The immunoglobulin superfamily protein Izumo is required for sperm to fuse with eggs. Nature.

[54] Fujihara Y (2012). SPACA1-deficient male mice are infertile with abnormally shaped sperm heads reminiscent of globozoospermia. Development.

[55] Shen Y (2019). Loss-of-function mutations in QRICH2 cause male infertility with multiple morphological abnormalities of the sperm flagella. Nat. Commun..

[56] Li YF (2010). CABYR isoforms expressed in late steps of spermiogenesis bind with AKAPs and ropporin in mouse sperm fibrous sheath. Reprod. Biol. Endocrinol..

[57] Yuan S (2015). Spata6 is required for normal assembly of the sperm connecting piece and tight head-tail conjunction. Proc. Natl Acad. Sci. USA.

[58] Wang X, Wei Y, Fu G, Li H, Yu L (2015). Tssk4 is essential for maintaining the structural integrity of sperm flagellum. Mol. Hum. Reprod..

[59] Xu B (2008). Targeted deletion of Tssk1 and 2 causes male infertility due to haploinsufficiency. Dev. Biol..

[60] Geyer CB (2009). A missense mutation in the Capza3 gene and disruption of F-actin organization in spermatids of repro32 infertile male mice. Dev. Biol..

[61] Wang H (2013). One-step generation of mice carrying mutations in multiple genes by CRISPR/Cas-mediated genome engineering. Cell.

[62] Sha QQ (2018). CNOT6L couples the selective degradation of maternal transcripts to meiotic cell cycle progression in mouse oocyte. EMBO J..

[63] Jiang Y, Zhang HY, Lin Z, Zhu YZ, Fan HY (2020). CXXC finger protein 1-mediated histone H3 lysine-4 trimethylation is essential for proper meiotic crossover formation in mice. Development.

[64] Liu C (2017). Sirt1 regulates acrosome biogenesis by modulating autophagic flux during spermiogenesis in mice. Development.

[65] MacLean B (2010). Skyline: an open source document editor for creating and analyzing targeted proteomics experiments. Bioinformatics.

