# Supplementary material for: Loss of CEP70 function affects acrosome biogenesis and flagella formation during spermiogenesis
Source: Cell Death Dis. 2021 May 12;12(5):478. doi: 10.1038/s41419-021-03755-z (PMC8116340; doi:10.1038/s41419-021-03755-z)
Supplement: Supplementary file 3 — Supplementary Materials [file 41419_2021_3755_MOESM3_ESM.docx]

Supplementary Materials for

**Loss of CEP70 function affects acrosome biogenesis and flagella formation during spermiogenesis**

Qiang Liu^1,2,3,4†^, Qianying Guo^1,2,3,4†^, Wei Guo^1,2,3,4^, Shi Song^1,2,3,4^, Nan Wang^1,2,3,4^, Xi Chen^1,2,3,4^, Andi Sun^1,2,3,4^,, Liying Yan^1,2,3,4^*, Jie Qiao^1,2,3,4,5^*

† indicate the authors contributed equally to the paper.

*Correspondence: jie.qiao@263.net; yanliyingkind@aliyun.com.


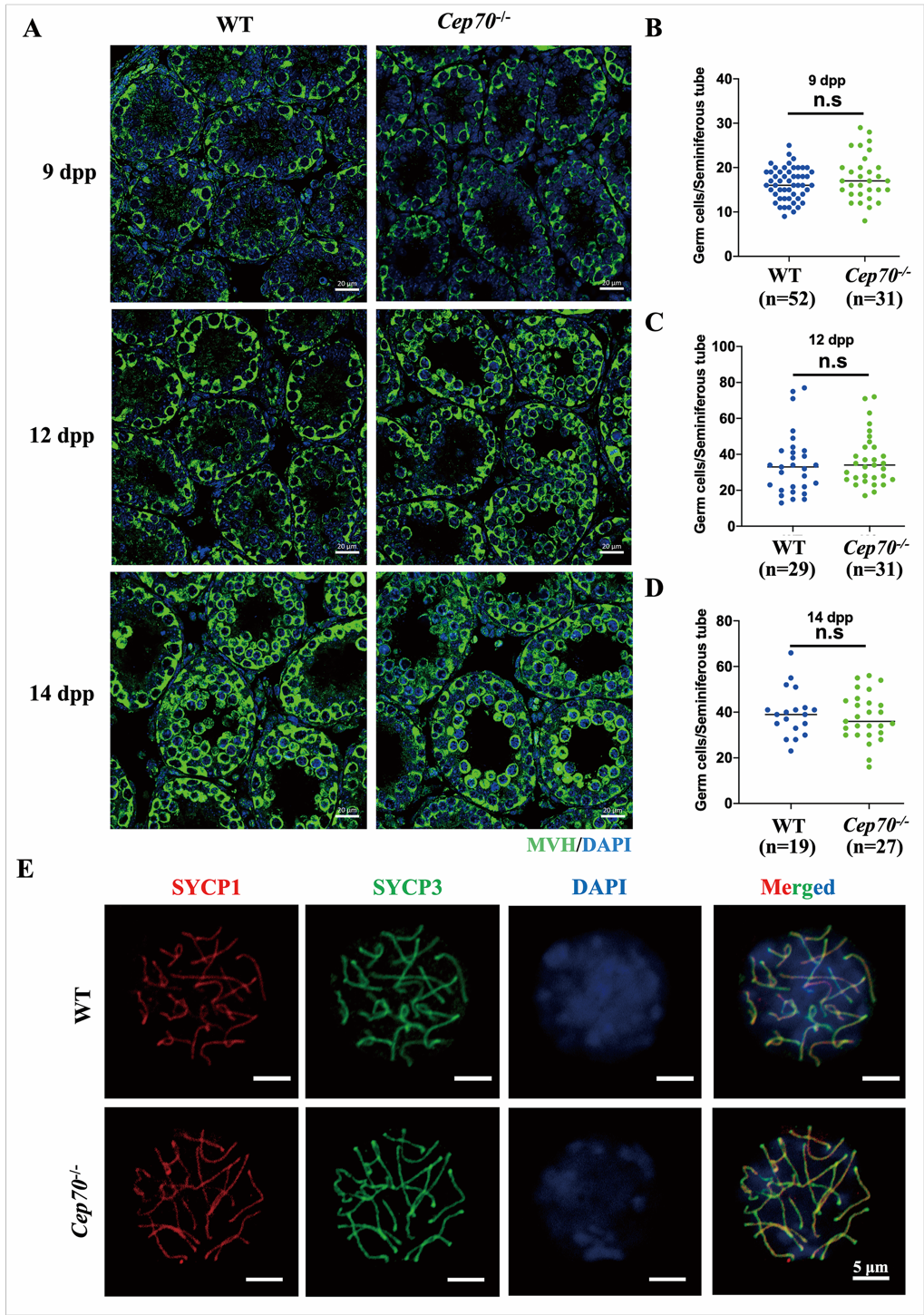


fig. S1. The prophase of meiosis I was not affected in *Cep70*^-/-^ male mice.

**(A)** MVH, a marker of germ-cells, immunofluorescence staining (green) of testes sections showed three key time points of the prophase of meiosis I, during the first wave of spermatogenesis in the WT and *Cep70*^-/-^ mice. DNA was counterstained with DAPI (blue). Scale bar: 20 μm. **(B-D)** Quantification of germ cells in each seminiferous tubule of the WT and *Cep70*^-/-^ mice at 9 dpp (n = 52 and n = 31, respectively), 12 dpp (n = 29 and n = 31, respectively), and 14 dpp (n = 19 and n = 27, respectively). Data are presented as the mean ± SD, n.s. not statistically significant. **(E)** Immunofluorescent co-staining of SYCP1 (red), SYCP3 (green), and DAPI (blue) in testes of the WT and *Cep70*^-/-^ mice. Scale bar: 5 μm.


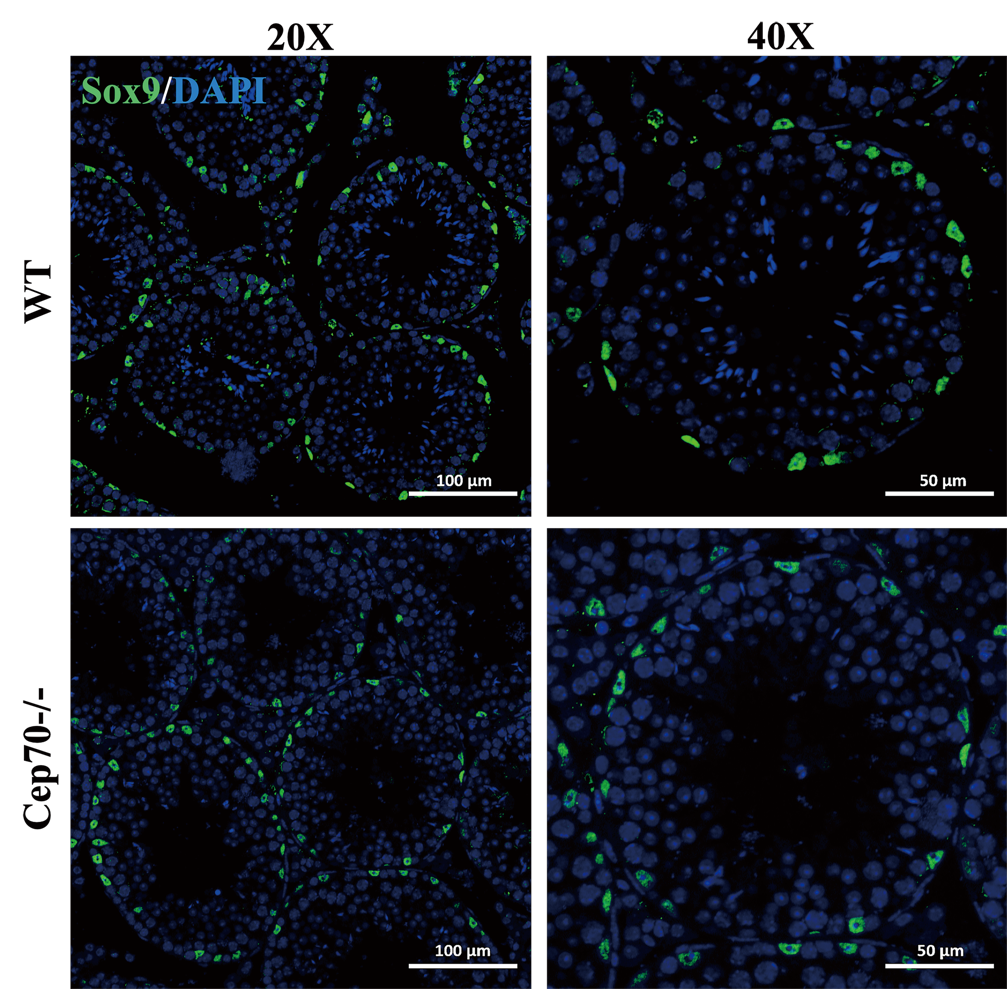


fig. S2. Sertoli cells were not affected in *Cep70*^-/-^ male mice.

Immunofluorescence staining of SOX9 (green), a marker of Sertoli cells, in sections of seminiferous tubules from WT and *Cep70*^-/-^ male mice. Scale bar: 100 μm (left) and 50 μm (right). The DNA was counterstained with DAPI (blue).


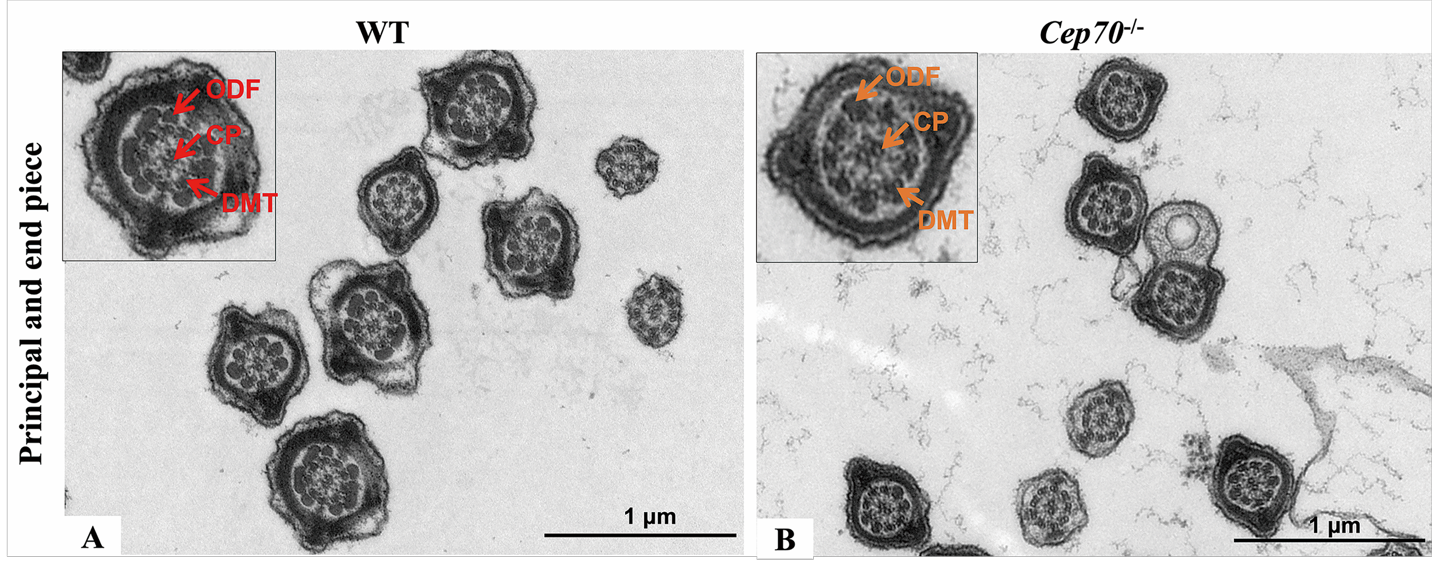


**fig. S3. TEM analysis of the WT and *Cep70*^-/-^ male mice sperm flagella.**

TEM analysis showed the normal ultrastructure of sperm flagellar cross-section (principal and end piece) in *Cep70*^-/-^ male mice. ODF, outer dense fiber; CP, central microtubules; DMT, peripheral microtubule doublets. Scale bar: 1 μm.


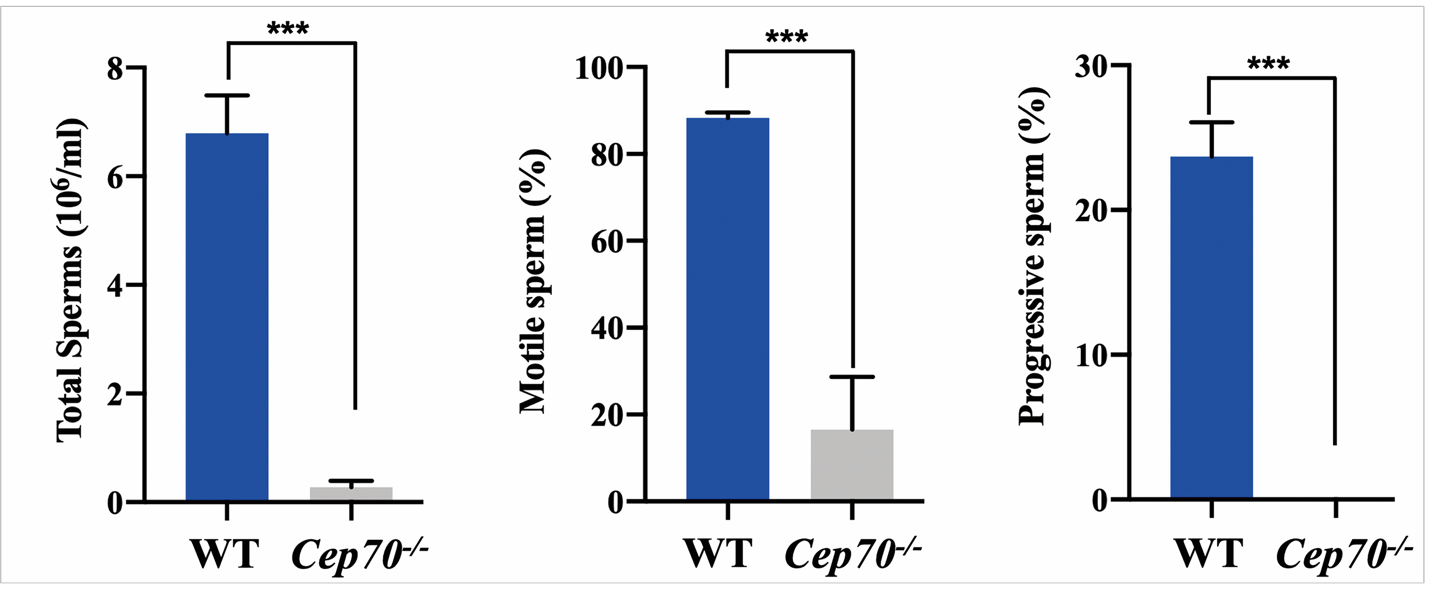


**fig. S4. Sperm motility assays of the WT and *Cep70*^-/-^ male mice by CASA.**

The amount of total sperm, the ratio of motile sperm, and the ratio of progressive sperm were significantly lower in *Cep70*^-/-^ male mice than in WT mice (n = 3). Data are presented as the mean ± SD, *p <* 0.001 (***).

Table S1. The sequence information of sgRNAs and genotyping primers

| sgRNAs sequence | |
| --- | --- |
| sgRNA1-F | TAGGAACAATCATCACAAAAAATG |
| sgRNA1-R | AAACCATTTTTTGTGATGATTGTT |
| sgRNA2-F | TAGGACGTCAGCAGAGCATGATAC |
| sgRNA2-R | AAACGTATCATGCTCTGCTGACGT |
| Genotyping primers | |
| Forward | CTTGTAGCAGGAGGAAGCAG |
| Reverse | AGTGGTTCACAATCATTCAT |

Table S2. The sequence information of sgRNAs and genotyping primers

| Target | Primer sequence（5’- 3’） | Product（bp） |
| --- | --- | --- |
| Exon 3-Forward | TTGCCCTGCAAGAGTAGTGG | 396 |
| Exon 3-Reverse | ACTAAATCCTCCCTTTGGAGACT |  |
| Exon 4-5-Forward | TGAGGCCTACATATGAGTATTCTGT | 627 |
| Exon 4-5-Reverse | AAGGCAGTTATTTTTAGAACGCA |  |
| Exon 6-Forward | TGCAAAGAAACTTTAGAAAGGCA | 360 |
| Exon 6-Reverse | CATGGAAGTCACCTGGGACC |  |
| Exon 7-Forward | TGTCTTGAATGCACGCAATTAT | 568 |
| Exon 7-Reverse | TGAGCAGGGAAAAACAATGCAG |  |
| Exon 8-Forward | AGAATTGTTGGGACAAGGAGGAT | 620 |
| Exon 8-Reverse | CAGGGGCTAGATCATATCAGTAGAA |  |
| Exon 9-Forward | AGCAGTGCTTTTTCACCTCCT | 586 |
| Exon 9-Reverse | AAGTTTCTCTCACCTGGTTTCC |  |
| Exon 10-Forward | ATCTGGATGCCTCACCAACT | 912 |
| Exon 10-Reverse | AGAGCAAAGGAGCTAGAATGGT |  |
| Exon 11-Forward | TCTAAATGGCCAGTAAACTCCA | 554 |
| Exon 11-Reverse | ACACCGCATGTTCTCACTCATAG |  |
| Exon 12-Forward | TATAAGGCTGTTGGGAGGGT | 358 |
| Exon 12-Reverse | TATGTCACGGAAAGACAAGACAGA |  |
| Exon 13- Forward | ACTGCAGATCTTGGGACTTG | 671 |
| Exon 13- Reverse | GGGAAGGGCCTAGAAGTACA |  |
| Exon 14-Forward | AAGCCTGTGTTTACCTGGAGAA | 568 |
| Exon 14-Reverse | TGGCATATTGCTGTCCTGTCC |  |
| Exon 15-16-Forward | TGAACGGTCACCTTCTAATGTT | 817 |
| Exon 15-16-Reverse | TCACCCCTCCAGGAACTCTC |  |
| Exon 17-Forward | GCTCAGAAAGAAAAGACTGTAGTAA | 514 |
| Exon 17-Reverse | GACAATGACAGAGCCAGGGT |  |
| Exon 18-Forward | TAAACAGAGCCCCTGTGAAGC | 654 |
| Exon 18-Reverse | AGAGAGCCCTTGGCATTCCT |  |
